# Prostaglandin E_2 _metabolism in rat brain: Role of the blood-brain interfaces

**DOI:** 10.1186/1743-8454-5-5

**Published:** 2008-03-04

**Authors:** Eudeline Alix, Charlotte Schmitt, Nathalie Strazielle, Jean-François Ghersi-Egea

**Affiliations:** 1 INSERM, U 842, Lyon; Université de Lyon; Faculté de médecine Laennec, UMR-S842, Lyon, F69372, France; 2 Brain-*i*, 34 Rue du Dr Bonhomme, Lyon, F69008, France

## Abstract

**Background:**

Prostaglandin E_2 _(PGE_2_) is involved in the regulation of synaptic activity and plasticity, and in brain maturation. It is also an important mediator of the central response to inflammatory challenges. The aim of this study was to evaluate the ability of the tissues forming the blood-brain interfaces to act as signal termination sites for PGE_2 _by metabolic inactivation.

**Methods:**

The specific activity of 15-hydroxyprostaglandin dehydrogenase was measured in homogenates of microvessels, choroid plexuses and cerebral cortex isolated from postnatal and adult rat brain, and compared to the activity measured in peripheral organs which are established signal termination sites for prostaglandins. PGE_2 _metabolites produced *ex vivo *by choroid plexuses were identified and quantified by HPLC coupled to radiochemical detection.

**Results:**

The data confirmed the absence of metabolic activity in brain parenchyma, and showed that no detectable activity was associated with brain microvessels forming the blood-brain barrier. By contrast, 15-hydroxyprostaglandin dehydrogenase activity was measured in both fourth and lateral ventricle choroid plexuses from 2-day-old rats, albeit at a lower level than in lung or kidney. The activity was barely detectable in adult choroidal tissue. Metabolic profiles indicated that isolated choroid plexus has the ability to metabolize PGE_2_, mainly into 13,14-dihydro-15-keto-PGE_2_. In short-term incubations, this metabolite distributed in the tissue rather than in the external medium, suggesting its release in the choroidal stroma.

**Conclusion:**

The rat choroidal tissue has a significant ability to metabolize PGE_2 _during early postnatal life. This metabolic activity may participate in signal termination of centrally released PGE_2 _in the brain, or function as an enzymatic barrier acting to maintain PGE_2 _homeostasis in CSF during the critical early postnatal period of brain development.

## Background

Prostaglandin E_2 _(PGE_2_) is a main product of the cyclooxygenase (Cox) pathway. Two Cox isoenzymes, Cox-1 and Cox-2, convert arachidonic acid released by phospholipases A_2 _to PGH_2_, which in turn is metabolized by terminal prostaglandin E synthases into PGE_2 _[1]. While Cox-1 is constitutively expressed in most tissues where it fine-tunes physiological processes [2], Cox-2 expression is very limited in normal conditions in peripheral organs. Yet it is induced by inflammatory stimuli, and then, being functionally coupled to microsomal prostaglandin E synthase 1, it plays a major role in the response to inflammation via PGE_2 _production [3].

In the brain, our current understanding of PGE_2 _metabolic cascade indicates that Cox-2 is constitutively expressed in several neuronal cell populations, especially in hippocampal and cortical glutamatergic neurons. The enzyme participates in synaptic activity, hippocampal long-term synaptic plasticity, and brain maturation (reviewed by Chen and Bazan, and Minghetti [4,5]). Inflammatory challenges trigger the cerebral upregulation of Cox 2, particularly in venule endothelial cells, and the subsequent production of PGE_2_. The prostaglandin acts as a modulator of the sickness behavior syndrome, and specifically induces fever via hypothalamic EP3 receptor activation [6,7]. The efficiency and length of the biological response to PGE_2 _is dependent upon the balance between its production and its inactivation. In peripheral organs, the first step of the inactivation process is mediated by NAD^+^-dependent 15-hydroxyprostaglandin dehydrogenase (PGDH) [8]. This enzyme is particularly active in the lung or the kidney. By contrast, it is considered absent in the brain of rodent and other mammalian species, from late gestation throughout postnatal life [1,9,10]. PGE_2 _catabolism has however been reported specifically in the choroid plexus of sheep during postnatal development and to some extent in adulthood [11]. Immunohistochemical evidence for the presence of PGDH in lamb choroid plexus exists also [12]. The choroidal tissue constitutes a major interface between the cerebrospinal fluid (CSF) and the blood, and in conjunction with the cerebral capillaries regulates the exchanges between the blood and the central nervous system. The mechanisms responsible for this crucial regulation are multiple and involve barrier and transport, as well as metabolic processes towards biologically active endogenous compounds.

We investigated in rats, whether the cells forming the blood-brain interfaces are a site of PGE_2 _metabolism into inactive compounds and as such, of signal termination. We isolated cerebral capillaries and choroid plexuses from developing and adult rat brain, measured PGDH activity in these tissues, and identified the metabolites actually produced from PGE_2_.

## Methods

### Reagents

PGE_2 _was purchased from Biomol International (Plymouth Meeting, PA, USA), 15-keto-PGE_2_, 13,14-dihydro-15-keto-PGE_2 _and β-nicotinamide adenine dinucleotide (NAD^+^) from Sigma (St Louis, MO, USA), bicyclo-PGE_2 _(11-deoxy-13,14-dihydro-15-keto-11β,16ε-cyclo-PGE_2_) from Cayman Chemical (Ann Arbor, MI, USA), and [^3^H]PGE_2 _(160 Ci/mmol) from Perkin Elmer Life Sciences (Boston, MA, USA). Bovine serum albumin and dextran used for capillary isolation were from I.D. Bio (Limoges, France), and Sigma, respectively. All other reagents were from high purity grades.

### Animals and tissue isolation

Animal care and procedures have been conducted according to the guidelines approved by the French Ethical Committee (decree 87–848) and by the European Community directive 86–609-EEC. Rats, 200–240 g, Sprague-Dawley males or timed pregnant females were obtained from Harlan, Gannat, France. Following halothane anesthesia and decapitation of the animals, rat brains were removed and the choroid plexuses were sampled intact under a stereomicroscope, briefly rinsed in Ringer-Hepes (RH) buffer [13], and kept at -80°C until used for enzymatic measurement. Kidney, lung and meninges-free brain cortex were also sampled. In some experiments freshly isolated intact choroid plexuses from both adult and 2-day-old rats were kept in RH buffer at 37°C for metabolic analysis. Microvessels from 9-day-old and adult brain cortices were isolated at 4°C in oxygenated buffers according to a previously described procedure [14], except that the capillaries were collected on a 40 μm-mesh nylon filter instead of glass beads. The purity of each preparation was controlled by phase contrast microscopy and by measuring the γ-glutamyl transferase specific activity as a capillary marker [14].

### Prostaglandin dehydrogenase activity measurement

Pools of choroid plexuses from at least eight 2-day-old or four adult animals, pools of isolated brain microvessels from twelve 9-day-old animals or four adults, brain cortex, kidney or lung tissue were homogenized in 50 mM Tris, 1 mM EDTA, 2 mM DTT buffer, pH 7.4, using a glass-glass homogeniser. The homogenates were centrifuged for 30 min at 14 000 rpm at 4°C, and the resulting supernatant assayed for PGDH activity. This measurement was performed at 37°C by kinetic analysis on a VARIAN Carry 100 double-beam spectrophometer (Mulgrave, VIC, Australia) set at 340 nm as follows: the supernatant was added to the Tris-EDTA-DTT buffer in both reference and sample cuvettes. After baseline stabilisation, NAD^+ ^(1 mM) was added to both cuvettes and the baseline further recorded until it stabilized again. PGE_2 _(20 μM) was then added to the sample cuvette and the optical density was recorded to follow the appearance of the reduced nucleotide NADH. The specific activity was calculated using the extinction coefficient of 6.22 × 10^-3 ^M^-1^.cm^-1^. An aliquot of kidney supernatant was run in each set of measurements as an internal control. The total protein content of the supernatants was determined by the method of Peterson [15] with bovine serum albumin as the standard.

### PGE_2 _metabolism by isolated choroid plexuses

Choroid plexuses from lateral and fourth ventricles were treated separately. Choroid plexuses from four 2-day-old animals or two adult animals were pooled and incubated on a rotating shaker in 160 μl of RH at 37°C for 5 or 45 min in the presence of 100 nCi of [^3^H]PGE_2_. Incubation medium without tissue was run in parallel with each set of measurements. At the end of the incubation, the choroidal tissue was removed and added to 40 μl of distilled water, homogenized in the presence of an additional 40 μl of acetonitrile, and centrifuged at 14,000 rpm for 30 min. The resulting supernatant and the incubation medium were then analyzed by HPLC. Choroid plexus protein content was evaluated separately on pools of choroid plexus tissue from the same litter (2-day-old animals), or from the same batch of animals (adults).

### HPLC analysis, and expression of the results

Incubation media and homogenate supernatants were analysed by reverse phase HPLC performed on a LC10 Shimadzu system (Duisburg, Germany) as follows: Samples (20 or 40 μl) were loaded with a mix of unlabelled PGE_2 _and its metabolites to allow UV detection, applied onto an Ultrasphere ODS RP-18 analytical column (5 μm, 46 mm × 150 mm, Beckman, Fullerton, California, USA), and eluted using a mobile phase of 35% acetonitrile/0.1% acetic acid/water pumped at 1 ml/min. Absorbance of the effluent was monitored at 210 nm. The effluent was collected for radiochemical analysis by liquid scintillation counting. Retention times of PGE_2_, 15-keto-PGE_2_, 13,14-dihydro-15-keto-PGE_2_ and bicyclo-PGE_2_ were 8.5, 12, 16 and 43 min, respectively. The purity of radiolabelled PGE_2_ was estimated from the incubation medium without choroidal tissue, as the ratio of radioactivity associated with PGE_2_ to the total radioactivity recovered and was taken into account in further calculations. Amounts of remaining PGE_2_ and of the metabolites produced during the incubation with the choroidal tissue were expressed as percentage values of the initial PGE_2_-associated radioactivity. The radioactive profile obtained from the incubation medium without tissue was used as a background profile. The background radioactivity eluted within the time-frame of collection for each metabolite was subtracted from the corresponding radioactivity measured in incubation medium following tissue metabolism. The amount of radioactivity (nCi) associated with PGE_2_ and each metabolite was calculated separately for the medium and the choroidal tissue, and then summed to generate the total % of PGE_2_ remaining, or metabolite produced at the end of the incubation period. To establish the tissue-medium distribution of PGE_2_ and 13,14-dihydro-15-keto-PGE_2_, the amount of each species present in the incubation medium and in the choroidal tissue at the end of the incubation was expressed as % of the total amount (medium plus tissue).

## Results

### 15-hydroxyprostaglandin dehydrogenase specific activity at blood-brain interfaces

PGDH activity was measured in homogenates of choroid plexuses, brain microvessels and cortical tissue (Table 1). The enzymatic analysis confirmed the absence of a detectable PGE_2 _metabolism in cerebral cortex of both adult and 2-day-old animals, and indicated that adult cerebral microvessel preparations also lack PGDH activity. The earliest postnatal period allowing pure capillary isolation is day 9 which marks the end of the sprouting period during which the basal lamina of primitive vessels remains thin and uneven [16]. The activity measured in microvessel preparations from 9-day-old animals was also undetectable (not listed in Table 1). By contrast, this enzymatic activity was readily detected in choroidal material sampled from lateral or fourth ventricle of 2-day-old rat brain. In both types of choroid plexus the activity was lower than in kidney and lung, two peripheral organs involved in prostaglandin signal termination (p < 0.01 and p < 0.05, respectively, one-way ANOVA followed by Tukey-Kramer's test). It strongly decreased in adult choroid plexus (p < 0.05, one-tailed student's t-test for unequal variance).

**Table 1:** Prostaglandin dehydrogenase specific activity in tissues from 2-day-old and adult rats.

	2-day-old	Adult
Lung	1.64 ± 0,42 (5)	0.47 ± 0,08 (5)
Kidney	2.29 ± 0,23 (7)	0.93 ± 0,16 (6)
Cerebral cortex	ND	ND
Fourth ventricle choroid plexus	0.10 ± 0,02 (3)	0.027 ± 0,012 (6)
Lateral ventricle choroid plexus	0.19 ± 0,05 (3)	0.025 ± 0,018 (5)
Cerebral microvessels	na	ND

Data (in nmol.mg protein^-1^.min^-1^) are expressed as mean ± SE (n). ND: not detectable. na: not available (see text). The detection limit was calculated as being 0.02 for microvessel and choroidal preparations, and 0.01 for cerebral cortex.

### PGE_2 _metabolism in choroid plexuses

**Figure 1:**
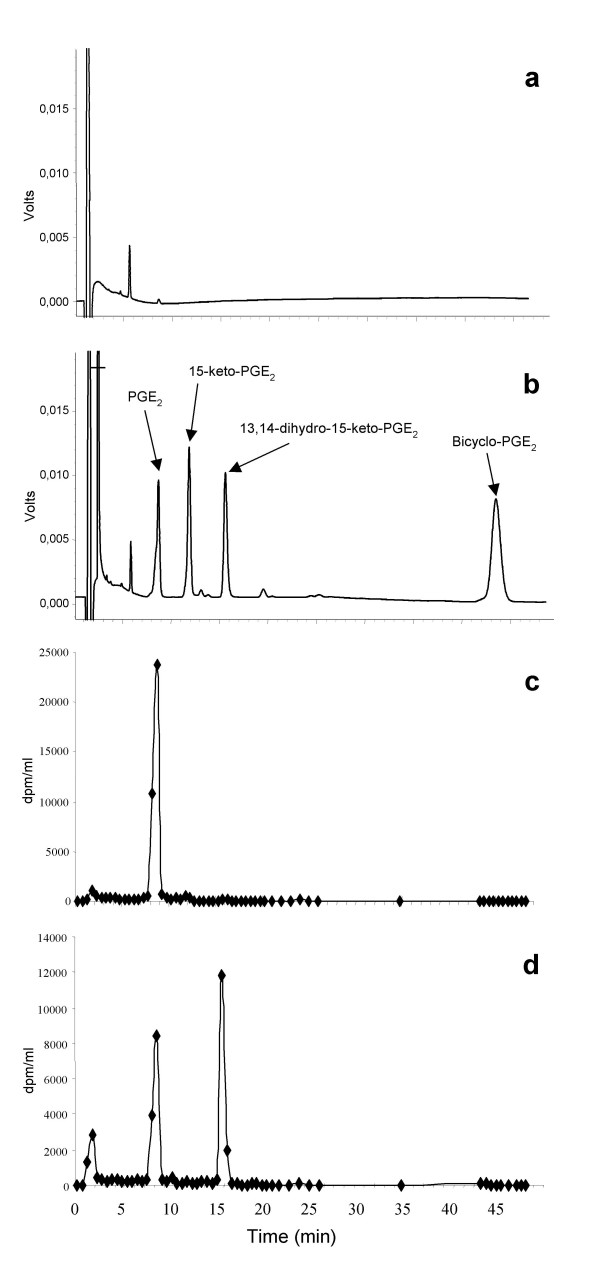
**Radiochemical reverse phase HPLC analysis of PGE_2 _metabolism by isolated choroid plexuses**. Typical chromatograms are shown: a and b are UV profiles of RH buffer (a) and RH buffer supplemented with PGE_2 _and its main potential metabolites (b); c and d are radiochemical chromatograms of RH medium incubated for 45 min in the absence (c) and presence (d) of lateral ventricle choroid plexuses from 2-day-old rats. In c and d the y axis represents radioactivity concentration (dpm/ml) in each collected fraction.

To investigate further the ability of the choroidal tissue to inactivate PGE_2_, we analyzed the metabolites produced upon exposure of intact isolated choroid plexus to the prostaglandin. Radiolabelled PGE_2 _was used as substrate and the metabolites were quantified by HPLC coupled to radiochemical detection. Figures 1a and 1b show the spectrophotometric profiles obtained using incubation medium supplemented or not, with a mix of unlabelled PGE_2 _and its main metabolites. The chromatographic conditions were adequate to separate the two potential PGE_2 _metabolites, i.e. 15-keto-PGE_2 _and 13,14-dihydro-15-keto-PGE_2_, as well as a non enzymatic cyclization breakdown product of 13,14-dihydro-15-keto-PGE_2 _known as bicyclo-PGE_2 _[11]. Typical radioactive profiles obtained by incubating [^3^H]PGE_2 _in the absence or presence of isolated intact lateral ventricle choroid plexuses of 2-day-old rats are shown in Figure 1c and 1d, respectively. In addition to the above-mentioned metabolites, an additional peak appeared with a short retention time (Peak 1; 1.75 min). Because the biotransformation of PGE_2 _into 15-keto-metabolites led to the release, as tritiated water, of one of the seven tritiated hydrogen atom on the prostaglandin, it is likely that tritiated water contributed to the radioactivity in this peak.

**Figure 2:**
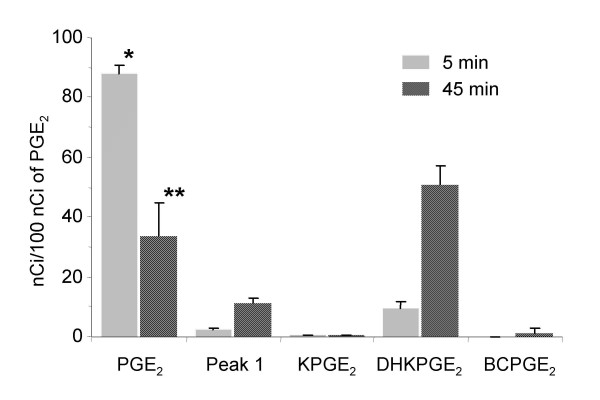
**PGE_2 _metabolism by isolated choroid plexuses from lateral ventricles of 2-day-old rat brain**. Data are expressed as mean ± SD of three different experiments, and represent the amount of radioactivity associated to each molecular species, relative to the initial radioactivity associated to PGE_2 _(see method), after either 5 or 45 minutes of incubation. * and **: different from PGE_2 _amount in incubation without choroidal tissue, p < 0.05 and 0.01, respectively, paired student's t-test. Abbreviations: KPGE_2_: 15-keto-PGE_2_, DHKPGE_2_: 13,14-dihydro-15-keto-PGE_2_, BCPGE_2_: bicyclo-PGE_2_.

The quantitative analysis of the data indicates that after 5 min incubation with lateral ventricle choroid plexuses from four 2-day-old animals, 12% of initial PGE_2 _has been metabolized, (representing a metabolic clearance of 18 μl, Figure 2). The most abundant metabolite, 13,14-dihydro-15-keto-PGE_2 _represents 9% of the radioactivity initially associated with PGE_2_. When the incubation time was extended to 45 min to allow substrate recycling, thus resulting in the biotransformation of 66% of PGE_2_, 13,14-dihydro-15-keto-PGE_2 _remained the main metabolite. The radioactivity associated with 15-keto-PGE_2 _and bicyclo-PGE_2 _at 45 min represented only 0.13 and 1% of the total radioactivity initially associated with PGE_2 _(Figure 2). At both 5 and 45 min, 75% of the amount of radioactivity forming Peak 1 may be accounted for by tritiated water formed during the transformation of PGE_2 _into keto-metabolites.

Very similar data were obtained using choroid plexuses sampled from the cerebral fourth ventricle of 2-day-old animals (not shown). The ability of intact isolated choroid plexuses from adult animals to inactivate PGE_2_, was also studied and compared to that of 2-day-old animals. The total amounts of PGE_2 _metabolized by lateral and fourth ventricle choroid plexuses of adults were respectively 10.5 and 6% of those biotransformed by the corresponding choroidal tissue of 2-day-old rats, following normalization for total protein content. As in 2-day-old material, the main metabolite identified was 13,14-dihydro-15-keto-PGE_2 _(data not shown).

### Location of PGE_2 _metabolite secretion by choroid plexuses

**Figure 3:**
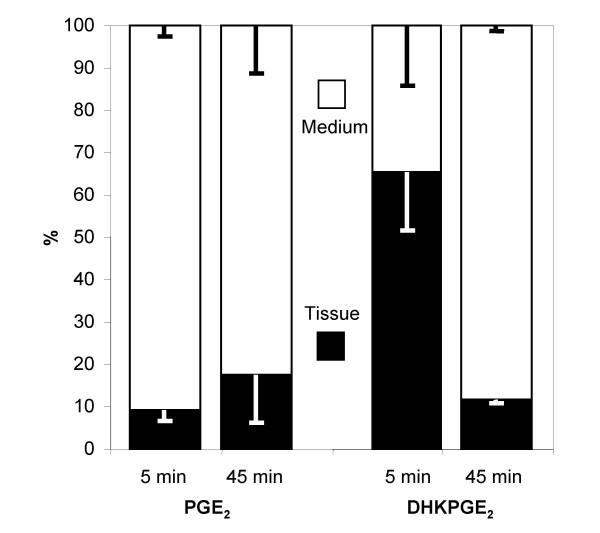
**Tissue-medium distribution of PGE_2 _and 13,14-dihydro-15-keto-PGE_2 _following exposure of 2-day-old rat lateral ventricle choroid plexuses to PGE_2_**. PGE_2 _and 13,14-dihydro-15-keto-PGE_2 _were quantified separately in the medium and in the choroidal tissue. The data are expressed as percentage of the total amount of molecule (either remaining PGE_2 _or produced 13,14-dihydro-15-keto-PGE_2_), at the end of the 5- and 45-minute incubation period. Mean ± SD, n = 3. Abbreviations as in Fig 2.

The medium and the choroidal tissue were analyzed separately to provide insight into the site of metabolite excretion from choroid plexus cells. The data are shown for lateral ventricle choroid plexuses (Figure 3). PGE_2 _remaining at the end of the incubation period of 5 min was mostly found in the incubation medium. By contrast, 13,14-dihydro-15-keto-PGE_2 _was mostly associated with the choroidal tissue, i.e. remained intracellular or concentrated within the stroma of the choroid plexus, rather than being excreted at the apical membrane of the epithelial cells. After 45 min, most of this metabolite was measured in the medium. The small amount of intermediate metabolite 15-keto-PGE_2 _produced remained associated with the tissue (not shown). Very similar data were obtained for fourth ventricle choroid plexus (not shown).

## Discussion

In this paper, we explored PGE_2 _catabolism in rat brain, focusing more specifically on the involvement of the blood-brain interfaces, at both postnatal and adult stages. PGDH is considered as a key oxidizing enzyme in PGE_2 _inactivation cascade, as the primary metabolite 15-keto-PGE_2_has a greatly reduced biological activity. 13,14-dihydro-15-keto-PGE_2 _is a secondary metabolite without biological activity generated by Δ13–15-keto-prostaglandin reductase [17].

In agreement with other groups, we observed no PGDH activity in brain cortical tissue in either young or adult animals, and we showed the absence of detectable enzyme in microvessels, i.e. at the blood-brain barrier. By contrast, and in accordance with data presented in sheep [12], we gathered evidence for PGE_2 _catabolic activity at the choroid plexus, which is the main site of the blood-CSF barrier. First, a significant specific activity of PGDH was measured in choroidal tissue homogenates prepared from 2-day-old rats. Second, the incubation of isolated whole choroid plexuses with PGE_2_, coupled to HPLC analysis, demonstrated the production of PGE_2 _metabolites, in particular 13,14-dihydro-15-keto-PGE_2_, thereby revealing the functional coupling of Δ13–15-keto-prostaglandin reductase to PGDH in the choroidal tissue.

The functional significance of this choroidal metabolic pathway may relate either to the termination of CSF-borne PGE_2 _signal, or to the prevention of blood-borne PGE_2 _penetration into the CSF. In our experimental setting for *ex-vivo *choroid plexus incubation, the isolated tissues were kept entire, which maximally limits rapid transfer between the external medium and the choroidal stromal core, and allows us to assume that most PGE_2 _was presented apically, i.e. at the CSF-side of the choroidal epithelium. Our results therefore suggest that *in vivo*, centrally released PGE_2 _that circulates in CSF will be metabolized to some extent by choroidal metabolizing enzymes. In line with this, an apical uptake of PGE_2 _mediated by an inwardly-directed probenecid-sensitive transport system has been reported in choroidal epithelium of different species [11,18,19]. Both metabolic and transport affinity constants have been determined in the micromolar range [8,20]. Given that PGE_2 _levels in the CSF remain below this concentration in physiological as well as pathological conditions [21-23], neither the enzymatic nor the transport process will reach saturation. The extent to which choroidal PGE_2 _metabolism can impact on the concentration of CSF-borne PGE_2 _remains however to be evaluated. Although PGDH activity is readily detected in choroidal tissue from young pups, it is 10 to 20 times lower than in kidney, or lung which is the main organ involved in peripheral signal termination of circulating prostaglandins [1]. In *ex-vivo *tissues from 2-day-old animals, after 5-min incubations, a significant amount of untransformed PGE_2_, similar to the total amount of metabolites produced, was found associated with the choroidal tissue. Although the precise localization of PGDH (epithelial and/or stromal) within the CP is unclear (see infra), the latter observation suggests that the metabolism capacity of the tissue towards PGE_2 _is lower than its uptake capacity. Transepithelial flux of PGE_2 _has been demonstrated in an *in vitro *model of the choroidal epithelium, implying that following its apical uptake from the CSF, native PGE_2 _can be exported across the basolateral stroma-facing membrane [19]. The relative capacity of this basolateral efflux mechanism, favoring PGE_2 _elimination in blood and thus supplementing the enzymatic signal termination mechanism needs to be established by comparison to uptake and metabolism in order to delineate how CSF-borne PGE_2 _concentration is controlled in developing animals.

PGE_2 _catabolism in the choroidal tissue may also be relevant in preventing blood-borne PGE_2 _from entering the CSF during early postnatal life. During this period, PGE_2 _is associated with hypothalamic maturation processes [24] and an increase in CSF PGE_2 _induces respiratory depression [reviewed in [11,12]]. Therefore abnormal blood PGE_2 _concentrations following infection or inflammation, were they to disrupt physiological PGE_2 _levels in CSF, could possibly lead to cerebral dysfunction. Based on the directionality and membrane distribution of the epithelial organic anion transporters that are likely candidates for membrane transfer of the prostaglandin [20], blood-to-CSF permeability to PGE_2 _is expected to be much lower than CSF-to-blood permeability. In 2-day-old rats, the metabolic activity of the choroid plexus tissue towards the prostaglandin will add an enzymatic barrier component to the transporter-mediated barrier properties of the epithelium, thereby contributing to buffer blood perturbations and maintain PGE_2 _homeostasis in CSF during this critical period of life. Of note, ontogenic maturation of the choroid plexuses is precocious and this tissue appears to play key functions in the control of brain homeostasis when the cerebral vasculature is still developing. [25,26]. The metabolic capacity displayed by the choroid plexuses towards PGE_2_, during the postnatal period highlights the early functional maturity of the interface.

In the adult, PGDH enzymatic activity is strongly decreased in choroidal tissue, a finding confirmed by the age-dependent decreased metabolic capacity observed in isolated choroid plexus. This leaves peripheral organs such as lung as the most likely sites of catabolism for the prostaglandin following its clearance from adult brain [1].

In *ex vivo *studies using short duration incubation, PGE_2 _metabolites were mostly associated with the choroidal tissue, indicating that they were produced by the epithelial cells and then preferentially released in the stroma, and/or produced by the fibroblasts or other stromal cells. When the incubation was prolonged, 13,14-dihydro-15-keto-PGE_2 _reached the external medium, probably as a result of diffusion from stroma. We previously showed, using a polarized cellular model of the blood-CSF barrier, that 13,14-dihydro-15-keto-PGE_2 _was produced and excreted at the basolateral membrane of the epithelial cells [19]. The latter step may involve the multidrug resistance associated protein abcc4, which transports organic anions such as prostaglandins [27], although its affinity for keto-metabolites remains to be established. This transporter has been immunodetected at the basolateral membrane of choroidal epithelium in several species [28]. PGE_2 _metabolism was however limited in the epithelial cells and did not significantly impede the transcellular flux of the prostaglandin [19]. Alternatively, the stromal hypothesis of PGE_2 _metabolism is supported by the immunohistochemical description in sheep of a switch in PGDH localisation from the epithelium to the stromal cells at birth [12]. In rat, attempts to locate PGDH in choroidal tissue by immunohistochemistry in our laboratory have been so far inconclusive (not shown). Regardless of the cellular site of PGE_2 _metabolism, the stromal i.e. blood side of metabolite excretion adds to the efficiency of the metabolic barrier by driving the clearance of the metabolite towards the blood circulation.

## Conclusion

Metabolism of PGE_2 _occurs in the rat choroid plexus tissue at early postnatal stage, leading to the production of the inactive 13,14-dihydro-15-keto-PGE_2 _metabolite. This function of the choroidal tissue, which disappears in adulthood, may be involved in maintaining CSF PGE_2 _homeostasis during the critical early phase of postnatal brain development.

## Competing interests

The authors declare that they have no competing interests.

## Authors' contributions

Data collection was performed by EA and CS. The study was conceived, designed and funded by JFGE and NS. NS realized the microdissections and helped to draft and revise the manuscript, and JFGE finalized the manuscript, and performed the statistical analyses.

All authors have read and approved the final version of the manuscript.

## References

[B1] Ivanov AI, Romanovsky AA (2004). Prostaglandin E2 as a mediator of fever: synthesis and catabolism. Front Biosci.

[B2] Smith WL, DeWitt DL, Garavito RM (2000). Cyclooxygenases: structural, cellular, and molecular biology. Annu Rev Biochem.

[B3] Consilvio C, Vincent AM, Feldman EL (2004). Neuroinflammation, COX-2, and ALS--a dual role?. Exp Neurol.

[B4] Chen C, Bazan NG (2005). Lipid signaling: sleep, synaptic plasticity, and neuroprotection. Prostaglandins Other Lipid Mediat.

[B5] Minghetti L (2004). Cyclooxygenase-2 (COX-2) in inflammatory and degenerative brain diseases. J Neuropathol Exp Neurol.

[B6] Konsman JP, Parnet P, Dantzer R (2002). Cytokine-induced sickness behaviour: mechanisms and implications. Trends Neurosci.

[B7] Romanovsky AA, Almeida MC, Aronoff DM, Ivanov AI, Konsman JP, Steiner AA, Turek VF (2005). Fever and hypothermia in systemic inflammation: recent discoveries and revisions. Front Biosci.

[B8] Ensor CM, Tai HH (1995). 15-Hydroxyprostaglandin dehydrogenase. J Lipid Mediat Cell Signal.

[B9] Nakano J, Prancan AV, Moore SE (1972). Metabolism of prostaglandin E 1 in the cerebral cortex and cerebellum of the dog and rat. Brain Res.

[B10] Pace-Asciake CR, Rangaraj G (1976). Prostaglandin biosynthesis and catabolism in the developing fetal sheep brain. J Biol Chem.

[B11] Krunic N, Adamson SL, Coceani F (2000). Differential uptake and catabolism of prostaglandin (PG)E(2) versus PGF(2alpha) in the sheep choroid plexus during development. Brain Res Dev Brain Res.

[B12] Krunic N, Adamson SL, Ackerley C, Okita RT, Coceani F (2000). Perinatal changes in choroidal 15-hydroxyprostaglandin dehydrogenase: implications for prostaglandin removal from brain. Brain Res Dev Brain Res.

[B13] Strazielle N, Ghersi-Egea JF (1999). Demonstration of a coupled metabolism-efflux process at the choroid plexus as a mechanism of brain protection toward xenobiotics. J Neurosci.

[B14] Ghersi-Egea JF, Leninger-Muller B, Suleman G, Siest G, Minn A (1994). Localization of drug-metabolizing enzyme activities to blood-brain interfaces and circumventricular organs. J Neurochem.

[B15] Peterson GL (1977). A simplification of the protein assay method of Lowry et al. which is more generally applicable. Anal Biochem.

[B16] Caley DW, Maxwell DS (1970). Development of the blood vessels and extracellular spaces during postnatal maturation of rat cerebral cortex. J Comp Neurol.

[B17] Tai HH, Ensor CM, Tong M, Zhou H, Yan F (2002). Prostaglandin catabolizing enzymes. Prostaglandins Other Lipid Mediat.

[B18] DiBenedetto FE, Bito LZ (1986). Transport of prostaglandins and other eicosanoids by the choroid plexus: its characterization and physiological significance. J Neurochem.

[B19] Khuth ST, Strazielle N, Giraudon P, Belin MF, Ghersi-Egea JF (2005). Impairment of blood-cerebrospinal fluid barrier properties by retrovirus-activated T lymphocytes: reduction in cerebrospinal fluid-to-blood efflux of prostaglandin E2. J Neurochem.

[B20] Strazielle N, Khuth ST, Ghersi-Egea JF (2004). Detoxification systems, passive and specific transport for drugs at the blood-CSF barrier in normal and pathological situation. Adv Drug Deliv Rev.

[B21] Aktan S, Aykut C, Ercan S (1991). Leukotriene C4 and prostaglandin E2 activities in the serum and cerebrospinal fluid during acute cerebral ischemia. Prostaglandins Leukot Essent Fatty Acids.

[B22] Engblom D, Saha S, Engstrom L, Westman M, Audoly LP, Jakobsson PJ, Blomqvist A (2003). Microsomal prostaglandin E synthase-1 is the central switch during immune-induced pyresis. Nat Neurosci.

[B23] Inoue W, Matsumura K, Yamagata K, Takemiya T, Shiraki T, Kobayashi S (2002). Brain-specific endothelial induction of prostaglandin E(2) synthesis enzymes and its temporal relation to fever. Neurosci Res.

[B24] Amateau SK, McCarthy MM (2004). Induction of PGE2 by estradiol mediates developmental masculinization of sex behavior. Nat Neurosci.

[B25] Dziegielewska KM, Ek J, Habgood MD, Saunders NR (2001). Development of the choroid plexus. Microsc Res Tech.

[B26] Strazielle N, Ghersi-Egea JF (2000). Choroid plexus in the central nervous system: biology and physiopathology. J Neuropathol Exp Neurol.

[B27] Reid G, Wielinga P, Zelcer N, van der Heijden I, Kuil A, de Haas M, Wijnholds J, Borst P (2003). The human multidrug resistance protein MRP4 functions as a prostaglandin efflux transporter and is inhibited by nonsteroidal antiinflammatory drugs. Proc Natl Acad Sci U S A.

[B28] Leggas M, Adachi M, Scheffer GL, Sun D, Wielinga P, Du G, Mercer KE, Zhuang Y, Panetta JC, Johnston B, Scheper RJ, Stewart CF, Schuetz JD (2004). Mrp4 confers resistance to topotecan and protects the brain from chemotherapy. Mol Cell Biol.

